# Attitude towards migration of psychiatric trainees and early career psychiatrists in Iran

**DOI:** 10.1186/s12909-021-02926-y

**Published:** 2021-09-22

**Authors:** Negin Eissazade, Dina Hemmati, Niloofar Ahlzadeh, Mohammadreza Shalbafan, Adeleh Askari-Diarjani, Homa Mohammadsadeghi, Mariana Pinto da Costa

**Affiliations:** 1 Student Research Committee, School of Medicine, Iran University of Medical Sciences, Tehran, Iran; 2 Mental Health Research Center, Psychosocial Health Research Institute, Department of Psychiatry, School of Medicine, Iran University of Medical Sciences, Tehran, Iran; 3 Clinical Research Development Unit, 22 Bahman Hospital, Qazvin University of Medical Sciences, Qazvin, Iran; 4 Institute of Psychiatry, Psychology & Neuroscience, King’s College London, London, UK; 5 Institute of Biomedical Sciences Abel Salazar, University of Porto, Porto, Portugal; 6 South London and Maudsley NHS Foundation Trust, London, UK

**Keywords:** Brain drain, Migration, Workforce, Health workers, Psychiatry, Job satisfaction, Work environment, Iran

## Abstract

**Introduction:**

Migration of medical professionals has been rapidly increasing in the past decades and it strongly affects origin and destination countries.

**Objectives:**

We aimed to explore the extent and the reasons of migration among psychiatric trainees and early career psychiatrists in Iran.

**Methods:**

Our semi-structured 61-items questionnaire inquired participants’ demographics, experiences of short-term mobility (from 3 months to 1 year), long-term migration (more than 1 year) and attitudes towards migration (current and future plans).

**Results:**

A total of 184 responses were received. Most (73.4 %) participants were female, and within the age range of 25–65 (Mean: 34.9). Only 15.2 % had a short-term mobility experience, mostly due to academic reasons (35.7 %). Most (75 %) stated that this short-term mobility experience influenced them in favor of migration. The majority (83.7 %) had ‘ever’ considered leaving Iran, and more than half (57.3 %) stated they ‘strongly agree’ or ‘agree’ to leaving the country ‘now’ (at the time of the study). The main reason to migrate from Iran was first political, followed by work, financial, social, religious, academic, and cultural reasons, and the least ranked were personal reasons. In relation to their 5-year plans, 67.3 % saw themselves in the country they currently live in, Iran. The main features reported for an attractive job were ‘pleasant work environment’ (97.3 %), ‘good welfare and social security’ (96.7 %) and ‘high salary ‘(96.2 %).

**Conclusions:**

This study calls for more support of psychiatric trainees and early career psychiatrists in Iran. Improvements in the political context, work conditions and finances might lower the rate of migratory intention and brain drain.

## Introduction

In 2017, the number of international migrants was reported as many as 258 million people. Migration to developed countries has been common among health professionals, and growing rapidly over the past decades [1, 2]. Discussions about the advantages and drawbacks of migration of health workers have been taking place in both origin and destination countries [3–5]. The reasons for migration have been termed as push and pull factors - and include social, financial, political, academic, cultural, personal or religious reasons - which can trigger health professionals to leave their homelands [6–8]. To address this, in 2010, the World Health Organization Global Code of Practice for the International Recruitment of Health Personnel was published by the World Health Assembly [9].

Patterns of medical staff migration, especially to developed nations, results in imbalanced human resources in both source and destination countries. These migration flows may weaken the donor health system; however, we should equally respect the human rights of health professionals for migration as in their homelands they lack resources and education opportunities [9]. Whilst migration is of interest to economists, labor markets, sociologists, and demographers, detailed information of health labor markets, salaries and unemployment rates should be public knowledge [10].

In upper income countries, as reported in 2014, there were 6.6 psychiatrists per 100,000 population and this number increased in 2017 to 11.9 psychiatrists per 100,000 population. In fact, around 70 % of the mental health professionals worldwide are working in high-income countries and about half of them working in Europe [11, 12]. Reports suggest that doctors from Asia commonly migrate to other continents. For example, India has a vast number of highly skilled health workers who have left the country [13]; in Pakistan, 20.3 % of the graduated physicians migrate from the country every year [14]; and China also reported increased rate of migration to America over the past 10 years among medical graduates [15].

Iran is the second largest country in the Middle East and one of the world’s oldest civilizations founded. According to the latest United Nations’ data, the current population of Iran is estimated 84,808,441 with the median age of 32.0 years old [16]. After the Islamic revolution in 1979, Iran went through major social, cultural, and religious changes, and based on the 2011 Iranian census, 99.98 % of Iranians believe in Islam, while only few of the population believe in Christianity, Judaism, and Zoroastrianism [17–19]. Mental health is strongly connected with each society’s culture and religious beliefs, which might impact both source and destination countries. In Iran, psychiatry training courses are available in more than 20 medical universities across the country [20]. Once medical students qualify as doctors, they can take the medical residency entrance examination and enter the 4-year training psychiatry field, if selected.

This article aimed to explore the attitudes towards migration among psychiatric trainees and early career psychiatrists in Iran and to identify the reasons behind migration.

## Methods

### Study design

This is a cross-sectional survey to explore migration trends in junior doctors in Psychiatry in Iran. This builds on the international study conducted in Europe by the European Federation of Psychiatric Trainees (EFPT) – the Brain Drain study [21] – using the same study instrument to assess this migration trends in Iran. This was a self-report, anonymous 61-item questionnaire inquiring about participants’ demographics, experiences of short-term (from 3 months to 1 year) mobility, long-term (more than 1 year) migration and trainees’ attitudes towards migration (current and future plans).

### Data collection

The questionnaire was sent by e-mail to approximately 700 psychiatric trainees studying in all the nationally recognized institutions in Iran, and to early career psychiatrists who are members of the Young Psychiatrists Section of Iranian Psychiatric Association, between March 2020 to March 2021. The inclusion criteria were to be a psychiatric trainee (i.e., a qualified physician who is undertaking psychiatric training in an institution in Iran) or an early career psychiatrist (i.e., a physician who has completed their psychiatric training not more than 5 years before). When in this manuscript the term ‘participants’ is used, this is in reference to both psychiatric trainees and early career psychiatrists.

### Data analysis

Data were analyzed by IBM SPSS Statistics (v. 25.0). To report the frequencies and percentages of categorical variables, descriptive statistics were used, and only valid percentages are reported. The demographic data and the variables of short-term mobility and long-term migration were compared using chi-square statistic tests.

## Results

One hundred eighty-four responses were received (response rate: 26.2 %). The majority of the participants were female (73.4 %) with an age range of 25–65 (Mean:34.9). The majority were married (65.2 %) and lived with their family (81.2 %). Most (64.7 %) were paid less than 250 €, and the wide majority (82.1 %) reported no additional income, with most (82.1 %) either ‘dissatisfied’ or ‘very dissatisfied’ with their income. Participants’ detailed socio-demographic data is presented in Table 1.

**Table 1:** Socio-demographic data of the participants

Variable	N (%)
**Gender**	
Male	49 (26.6%)
Female	135 (73.4%)
**Age**	25-65 (Mean:34.9)
**Marital status**	
Married	120 (65.2%)
Single	48 (26.1%)
In a relationship	11 (6.0%)
Separated	5 (2.7%)
**Children**	
Yes	59 (32.1%)
No	125 (67.9%)
**Living status**	
With family	148 (81.3%)
With friends	4 (2.2%)
Alone	32 (17.4%)
**Home-ownership status**	
Own a house	69 (37.5%)
Parents’ house	41 (22.3%)
Rented house	60 (32.6%)
Government property	16 (7.5%)
**Role in the health system**	
Psychiatry trainees	108 (58.7%)
Board-certified psychiatrists	42 (22.8%)
Non-board-certified psychiatrists	26 (14.1%)
Psychiatry fellows	8 (4.3%)
**Province**	
Tehran	76 (41.3%)
Mazandaran	6 (3.2%)
Golestan	2 (1%)
Guilan	12 (6.5%)
Markazi	2 (1%)
Isfahan	4 (2.1%)
Fars	9 (4.8%)
Sistan and Baluchestan	8 (4.3%)
Kermanshah	8 (4.3%)
Khorasan	13 (7%)
Hamedan	4 (2.1%)
Yazd	6 (3.2%)
Ghazvin	13 (7%)
Kerman	3 (1.6%)
Kermanshah	7 (3.8%)
Kordestan	3 (1.6%)
Khuzestan	3 (1.6%)
East Azarbaijan	2 (1%)
Zanjan	3 (1.6%)
**Income**	
< 250 €	72 (39.1%)
250-499 €	9 (4.8%)
500-999 €	2 (1%)
1000-1499 €	16 (8.6%)
1500-2000 €	13 (7%)
**Additional income**	
Yes	33 (17.9%)
No	151 (82.1%)
**Satisfaction with income**	
Very satisfied	1 (0.5%)
Satisfied	12 (6.5%)
Neither satisfied nor dissatisfied	20 (10.9%)
Dissatisfied	55 (29.9%)
Very dissatisfied	96 (52.2%)

Only 25 (13.5 %) had had previous short-term mobility experiences, which were mainly for education (48 %), and volunteer work (16 %). More than three-fourths (76 %) stated that this short-mobility had influenced them in favor of migration, with preferred destination countries: the UK, Germany, France, America, Italy, Syria, and Kuwait. Only a few (*N* = 13,7 %) had a migratory experience of more than a year. They had migrated to America, Canada, Britain, Syria, Georgia, and United Arab Emirates. They had either migrated with their families (*N* = 7, 53.8 %), partners (*N* = 5, 38.4 %) or with a friend (*N* = 1, 7.6 %). Among them, they were either ‘very satisfied’ (*N* = 5, 38.4 %), or ‘satisfied’ (*N* = 3, 23 %) with their experiences and the rest were ‘neither satisfied nor dissatisfied’ (*N* = 5, 38.4 %).

In relation to their 5 year-plan, most (*N* = 124, 67.3 %) answered they saw themselves in the country they already live in (Iran), 15.8 % (*N* = 29) stated ‘I have not made up my mind yet’, 9.8 % (*N* = 18) ‘Anywhere in the world’, 4.4 % (*N* = 9) ‘in Europe’, 1.1 % (*N* = 2) in America, and 1.1 % (*N* = 2) in Australia.

In relation to their migratory tendencies, the majority had ‘ever’ considered leaving Iran (*N* = 153, 83.7 %), but fewer had taken practical steps towards it (*N* = 51, 27.7 %) or had planned working in a different country (*N* = 66, 35.9 %).

In the Chi-square test, no significant correlations were found among the participant’s variables.

**Fig. 1:**
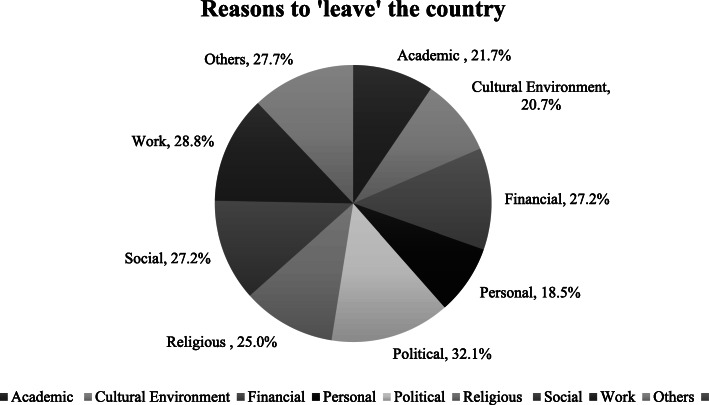
Reasons to ‘leave’ the country

**Fig. 2:**
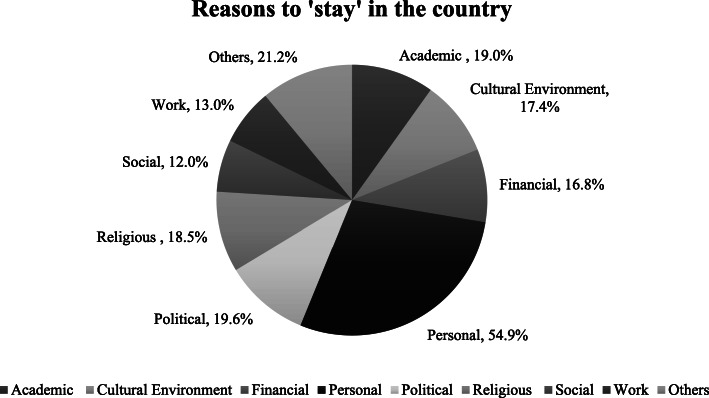
Reasons to ‘stay’ in the country

The main reason to ‘leave’ the country was political (32.1 %), followed by work (28.8 %) (Fig. 1). The main reason to ‘stay’ in the country was mainly due to personal reasons (54.9 %), followed by political reasons (19.6 %). The main conditions that participants required improvements in were financial (85.3 %), academic (64.1 %), and in their professional networks (63.5 %) (Fig. 2).

**Fig. 3:**
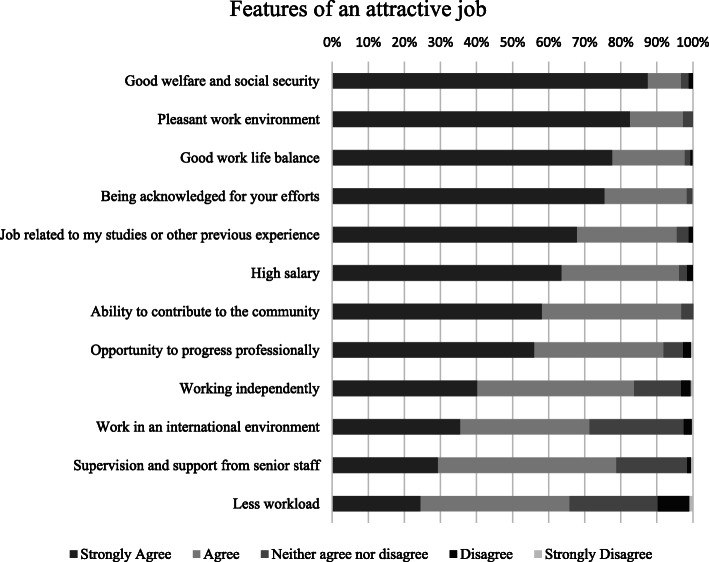
Features of an attractive job

The main features reported for an attractive job were ‘good welfare and social security’ (87.5 %), ‘pleasant work environment’ (82.6 %), and ‘good work life balance ‘(77.7 %) (Fig. 3).

## Discussion

### Key findings

All study participants were native Iranians and most of them were females (73.4 %). This is in line with the population of psychiatric trainees in Iran, of which 73.2 % are female doctors [20]. Participants mostly lived with their families, owned a house, were paid less than 250 €, and had no additional income. Not many of them had experienced mobility or migration. The majority (83.7 %) had ‘ever’ considered leaving Iran but only 27.7 % had taken practical steps towards migration. The main conditions recommended to be improved in Iran, were financial (85.3 %), academic conditions (64.1 %), and in their professional network (63.5 %). The main reasons to ‘stay’ in Iran were mainly personal (54.9 %), followed by political (19.6 %), and academic (19 %), whereas the top reasons to ‘leave’ Iran were political (32.1 %), work (28.8 %), and financial (27.2 %). In a 5-year perspective, 66.5 % planned on staying in Iran, 15.8 % had not decided yet, and 16.7 % planned on leaving the country. The main feature for an attractive job was reported ‘good welfare and social security’.

### Comparison with the literature

A study conducted in 2016, searching for ‘factors affecting the intention of migration among Iranian health workers, described that people who were most likely open to migration were under 35, had less than 5 years’ experience of work, had informal employment status, spoke more than one foreign language, had relatives living abroad and previous experience of mobility. The main reasons to migrate, among health workers, were seeking a better life, interdisciplinary discrimination, and wanting to have the experience of migration [22].

Comparing our findings in Iran, with the results from this study from 2013 to 2014 in 33 European countries, it was then reported that 13.3 % of the psychiatric trainees working in Europe were already immigrants (Switzerland, Sweden and UK being the top host countries). Two-thirds had ‘ever’ considered migration, and half were considering it at the time of the study. North and West countries were reported as the host countries and South and East as the donors. Academic, financial, and personal reasons triggered migration among psychiatric trainees in Europe [21].

Data from this study in Turkey [23], and the Baltics [24] showed that females demonstrated less tendency towards migration, whereas in Portugal it was reported to be slightly more [25]. In another study conducted in Germany, females who were in a relationship or had children were less likely to consider migration [26].

The main reasons to leave the country in Turkey were academic, work and financial [23], whereas in Portugal and in the Baltics were personal and financial, and in Spain were financial, work, and cultural [24, 25, 27]. In another study in Ireland, work and financial reasons were reported to be leading medical students to leave the country [28]. Similar to Iran, political, work, financial, and social conditions were reported as the main push factors.

In Romania, personal reasons, in Spain personal, cultural, and social reasons, and in Portugal, personal, academic, and work were the main reasons to stay in the country [25, 27, 29]. The main pull factors in Iran were reported personal, political, academic, and religious factors.

In a 5-year perspective, 57.6 % of the Estonian, Lithuanian and Turkish trainees [23, 24], 50.0 % Portuguese and Romanian trainees were considering working in another European country [25, 29]. In Iran, most of the participants in this study (66.8 %) saw their future-selves in the country they lived in, and the rest had not made up their minds yet (15.8 %), or saw themselves ‘anywhere in the world’ (9.8 %) or particularly ‘in Europe’ (4.4 %), America (1.1 %) or Australia (1.1 %).

### Strengths and limitations

This is the first study to investigate the attitudes of psychiatric trainees and early career psychiatrists in Iran towards migration. However, this study has some limitations. First, a low response rate (26.3 %) and a small sample size. Second, there are differences in the response rate among different provinces in Iran. Other limitations include the nature of the self-report data collection, the reporting bias and social-desirability bias (i.e., participants may answer questions in a manner that may be viewed favorably by others).

### Implications for practice, research, and policies

The findings of this study call for improvements in political, academic, work and financial conditions in Iran.

Lack of time and expertise in other fields, due to heavy healthcare work, leaves psychiatric trainees and early career psychiatrists with no time to look for an additional income, and dissatisfaction with their salary is considered a ‘push factor’. In addition, recent political variations and sanctions against Iran have led to imbalance in economy, bad welfare and social insecurity, lack of goods and medications, lack of academic facilities and have made transportation between countries harder. Rejecting to live under these influences and the desire to seek better opportunities and quality of life, the three ‘political, work, and financial’ reasons are on top of the list of push factors. In relation to work conditions, strong status, environment, better management, facilities, promotion and security are important factors.

This study recommends that psychiatric trainees and early career psychiatrists need to be supported and provided with efficient academic opportunities and facilities, higher salary and pleasant work place, which may decrease their willingness to migrate. Further investigation is needed to develop and implement a functional management plan.

## Conclusions

There is a significant willingness to migrate in psychiatric trainees and early career psychiatrists in Iran. Addressing their professional needs, and improve the political context, the work conditions, and their finances might lower the rate of migratory intention and brain drain. This study calls for more care and support for psychiatric trainees and early career psychiatrists in the healthcare system in Iran.

## Data Availability

The dataset used and analyzed during the current study can be shared by the corresponding author upon reasonable request.
